# A Threshold Logistic Modelling Approach for Identifying Thresholds between Antibiotic Use and Methicillin-Resistant *Staphylococcus aureus* Incidence Rates in Hospitals

**DOI:** 10.3390/antibiotics11091250

**Published:** 2022-09-15

**Authors:** Mamoon A. Aldeyab, Stuart E. Bond, Barbara R. Conway, Jade Lee-Milner, Jayanta B. Sarma, William J. Lattyak

**Affiliations:** 1 Department of Pharmacy, School of Applied Sciences, University of Huddersfield, Huddersfield HD1 3DH, UK; 2 Pharmacy Department, Mid Yorkshire Hospitals NHS Trust, Wakefield WF1 4DG, UK; 3 Institute of Skin Integrity and Infection Prevention, University of Huddersfield, Huddersfield HD1 3DH, UK; 4 Department of Microbiology, Mid Yorkshire Hospitals NHS Trust, Wakefield WF1 4DG, UK; 5 Scientific Computing Associates Corp., River Forest, IL 60305, USA

**Keywords:** antibiotic use, antibiotic resistance, antibiotic prescribing, antibiotic stewardship, threshold logistic modelling, thresholds, MRSA, epidemiology, clinical practice

## Abstract

The aim of this study was to demonstrate the utility of threshold logistic modelling, an innovative approach in identifying thresholds and risk scores in the context of population antibiotic use associated with methicillin-resistant *Staphylococcus aureus* (MRSA) incidence rates in hospitals. The study also aimed to assess the impact of exceeding those thresholds that resulted in increased MRSA rates. The study was undertaken in a 700-bed hospital in England between January 2015 and December 2021 (84 monthly observations). By employing the threshold logistic modelling approach, we: (i) determined the cut-off percentile value of MRSA incidence that defines a critical level of MRSA; (ii) identified thresholds for fluoroquinolone and co-amoxiclav use that would accelerate MRSA incidence rates and increase the probability of reaching critical incidence levels; (iii) enabled a better understanding of the effect of antibiotic use on the probability of reaching a critical level of resistant pathogen incidence; (iv) developed a near real-time performance monitoring feedback system; (v) provided risk scores and alert signals for antibiotic use, with the ability to inform hospital policies, and control MRSA incidence; and (vi) provided recommendations and an example for the management of pathogen incidence in hospitals. Threshold logistic models can help hospitals determine quantitative targets for antibiotic usage and can also inform effective antimicrobial stewardship to control resistance in hospitals. Studies should work toward implementing and evaluating the proposed approach prospectively, with the aim of determining the best counter-measures to mitigate the risk of increased resistant pathogen incidence in hospitals.

## 1. Introduction

Antimicrobial resistance (AMR) is a global threat to public health and economic development, contributing to increased morbidity, mortality, and healthcare costs with a significant impact on health systems [1,2,3,4,5]. In a recent comprehensive evaluation, the global burden associated with drug-resistant infections in 2019 was an estimated 4.95 million deaths, of which 1.27 million deaths were directly attributable to drug resistance [6]. Medicines are considered one of the key building blocks in health systems, and it is essential to make effective antimicrobial therapy accessible in order to support the sustainability of health systems [7,8]. However, inappropriate antibiotic prescribing practices and the emergence of AMR jeopardize access to effective antibiotic treatments [1,9,10,11,12,13,14,15,16].

Several studies have shown an association between antibiotic use and the subsequent development of resistance [17,18,19,20,21,22,23]. Antibiotic use (e.g., third-generation cephalosporins, fluoroquinolones, macrolides, and co-amoxiclav) has been linked to the development of methicillin-resistant *Staphylococcus aureus* (MRSA) in ecological population studies [24,25,26,27,28,29,30,31]. These studies applied time series techniques as a robust statistical method [24,25,26,27,28,29,30,31,32]. The value of time series analysis lies in its ability to measure and assess the effectiveness of different pathogen-controlling measures designed according to local antibiotic use, infection control practices, and resistance patterns [10,25]. While linear time series methods have been applied to determine the relationship between antibiotic use and resistance [24,25,26,27,28,29], studies suggest that non-linear relationships are more useful [19,20,30,31,33,34,35]. As a consequence of potential non-linear relationships, it was suggested that there might be a threshold of antibiotic use beyond which resistance would be triggered [19,20,33]. Recently, we developed a modelling concept, named threshold logistic, that improved the understanding of the impact of antibiotic use on AMR when use exceeds recommended thresholds. It can also provide targets for antibiotic consumption and a near real-time performance monitoring feedback system [36]. To demonstrate the utility of this modelling concept (i.e., threshold logistic), a Gram-negative pathogen (extended spectrum β-lactamase (ESBL)-producing *Escherichia coli* (*E. coli*)) was selected and the results of this evaluation were reported [36]. In this study, we aimed to demonstrate the utility of threshold logistic modelling methods in identifying thresholds for specific antibiotic use, and to understand the impact of exceeding thresholds of antibiotic use that result in increasing MRSA incidence. By employing a threshold logistic approach, we modelled the probability of a hospital reaching a critical level of MRSA in advance of such an event occurring. The benefit is an opportunity for the hospital to engage in counter-measures to mitigate the risk of an accelerated MRSA incidence rate. By using data from the same population for which we examined the incidence of ESBL-producing *E. coli* [36], we also provided tailored recommendations and an example of identifying antibiotic use targets for the management of Gram-positive and Gram-negative incidence rates in hospitals.

## 2. Results

**Figure 1:**
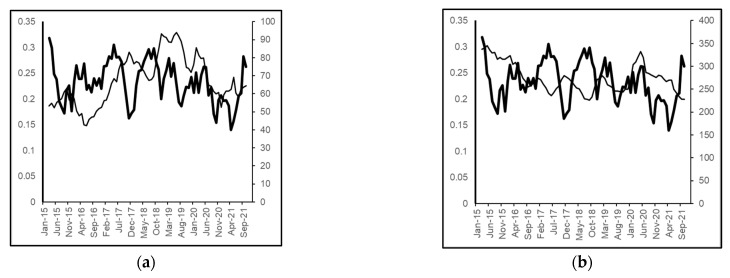
Monthly MRSA incidence versus use of selected antibiotic classes (thick line, MRSA, no. of cases/1000 OBD, 5-month moving averages, left-hand *y*-axis; thin line, antimicrobial use, DDD/1000 OBD, 5-month moving averages, right-hand *y*-axis). (**a**) fluoroquinolones and (**b**) co-amoxiclav.

Over the study period, 395 non-duplicated MRSA cases were identified. The average monthly MRSA incidence rate was 0.235 cases/1000 occupied bed-days (OBD) (range: 0.05–0.57). The average fluoroquinolone (predominantly levofloxacin and ciprofloxacin) use was 67.2 DDD/1000 OBD (range: 37.4–123.0), and the average co-amoxiclav use was 275.5 DDD/1000 OBD (range: 190.5–413.4). A graphical representation of the relationship between the identified antibiotics and the incidence of MRSA cases is shown in Figure 1.

### 2.1. Defining a Critical Level of MRSA Incidence Rates

A threshold logistic regression search algorithm was employed to identify the cut-off percentile value of MRSA incidence that we established as a high incidence rate. The maximization of classification sensitivity and specificity was used to select the percentile of MRSA incidence.

### 2.2. Threshold Logistic Method

**Figure 2:**
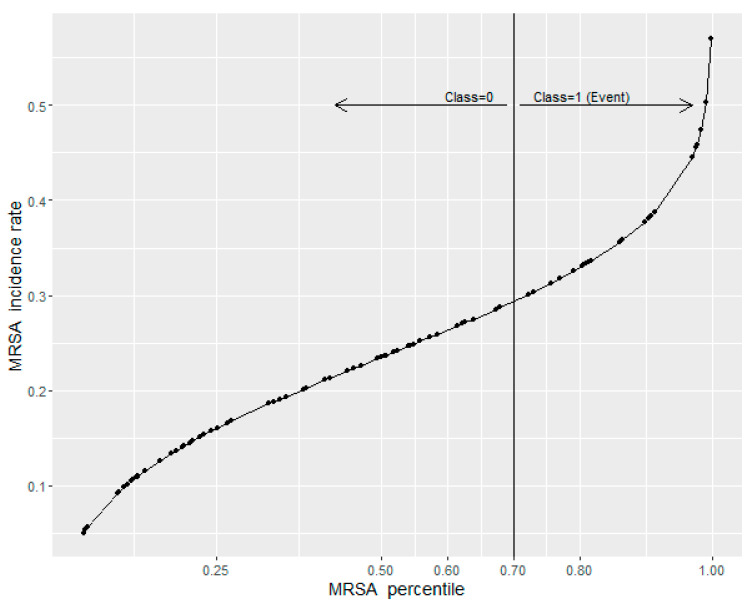
The empirical cumulative distribution function for MRSA historical data. The solid vertical line represents the 70th percentile (0.276 cases/1000 OBD).

Based on the search results, the 70th percentile (0.276 cases/1000 OBD) of MRSA incidence was selected as the cut-off in defining the dichotomous binary classification variable (Figure 2).

The variable representing the COVID-19 period was found to be insignificant (coefficient = −0.378; *p* = 0.572), therefore, it was removed from the models to maintain parsimony. Using threshold logistic models, fluoroquinolones were found to have a threshold at 55.96 DDD/1000 ODB, and co-amoxiclav was found to have a threshold at 312.19 DDD/1000 ODB (Table 1).

The threshold logistic analysis demonstrated that for every one-unit increase in fluoroquinolone and co-amoxiclav use above 55.96 and 312.19 DDD/1000 OBD, the average odds of an MRSA incidence rate exceeding the 70th percentile of historical levels increased by 4.98% and 5.05%, respectively (Table 1).

**Figure 3:**
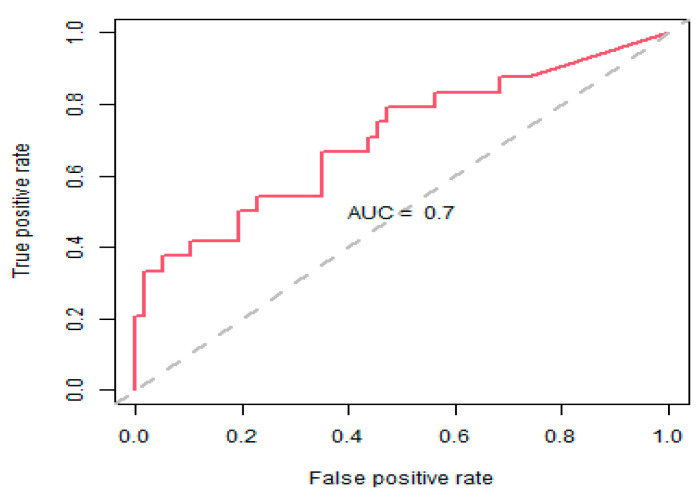
Receiver operator characteristic (ROC) chart showing the true positive classification rate against the false positive classification rate at different probability cut-off thresholds. The area under the curve (AUC) is an aggregate measure of performance across all possible classification thresholds.

**Figure 4:**
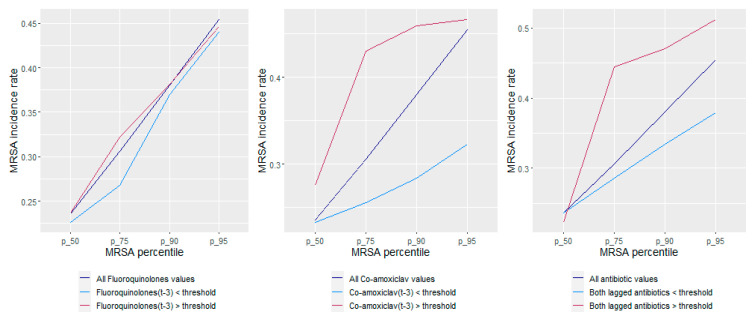
Cumulative MRSA incidence rates relative to fluoroquinolone and co-amoxiclav use being above or below their respective thresholds.

The classification accuracy for this model was 77.8% and the area under the curve (AUC) measure for the receiver operator characteristic (ROC) curve was 70% (Figure 3). Cumulative MRSA incidence rates, in relation to fluoroquinolone and co-amoxiclav use being above or below their respective thresholds, are presented in Figure 4. MRSA incidence rates were consistently higher when antibiotic thresholds were exceeded, and lower when thresholds were kept below their defined targets (Figure 4).

**Figure 5:**
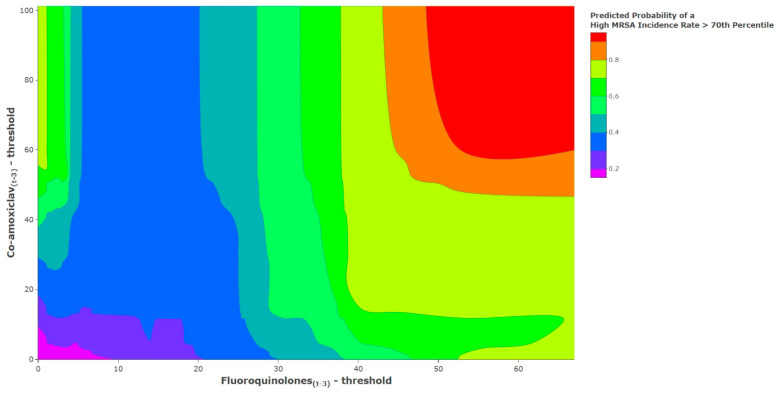
Illustrations of the associations between antibiotic use above their identified thresholds and the predicted probability of exceeding the 70th percentile of historical MRSA incidence rates using the identified threshold logistic model.

The contour chart shows the results of triangulating antibiotic unit changes above the identified thresholds using the threshold logistic model with the predicted probability of exceeding the 70th percentile of the historical MRSA incidence rate (Figure 5). We observed increases in the probabilities of reaching a high MRSA incidence rate as the combined levels of antibiotic use increased. The *x*-axis is the threshold-adjusted fluoroquinolone use at lag 3, and the *y*-axis is the threshold-adjusted co-amoxiclav use at lag 3. The lower-left corner of the plot is the point at which both antibiotic series are equal to their identified thresholds; co-amoxiclav_(t−3)_ = 312.19 DDD/1000 OBD, and fluoroquinolone_(t−3)_ = 55.96 DDD/1000 OBD. It is the point at which the basis functions evaluate to 0. The basis functions are:

$$Basis\;Function\;for\;co-amoxiclav=max\left((co-amoxiclav{\;}_{\left(t-3\right)}-312.19),\;0\right)$$

$$Basis\;Function\;for\;fluoroquinolone=max\left((fluoroquinolone_{\left(t-3\right)}-55.96),\;0\right)$$

### 2.3. Risk Scores 

Table 2 shows the ongoing performance for 2021 and presents risk scores that were generated from the threshold logistic model for MRSA incidence rates exceeding the 70th percentile (0.276 cases/1000 OBD). Three alert signal levels (Low, Medium, and High) were devised for coding the probability risk scores (Table 2).

To aid the understanding of Table 2, and taking January 2021 as an example, the MRSA incidence rate was above 0.276 (70th percentile). Fluoroquinolone use was above 55.96 DDD/1000 OBD three months prior, which recodes the basis function for fluoroquinolone use to 2.29. Co-amoxiclav use was below 312.19 DDD/1000 OBD three months prior, which recodes the basis function for co-amoxiclav use to 0. Applying these values to the threshold logistic model, a predicted MRSA incidence probability above the 70th percentile of 0.148 was produced, translating to a Low alert signal.

The overall classification accuracy, which was based on the coded alert signals, is shown in Table 3. Our analysis showed that a Low alert signal was correct 28 out of 33 times in identifying an MRSA incidence rate below the 70th percentile (a 5.6 to 1 accuracy ratio). A High alert signal was correct 13 out of 26 times in identifying MRSA incidence rates above the 70th percentile (a 1 to 1 accuracy ratio). For the Medium alert signal, we were more than two times as likely to be below the 70th percentile of the MRSA incidence rate.

### 2.4. What-If Scenarios

Predictive models with varied lag structure are conducive to performing a “what-if” scenario by adjusting the expected antibiotic levels and observing the change in predicted outcomes. In this model, although both identified antibiotics (i.e., fluoroquinolone and co-amoxiclav) were lagged, they were both entered into the model three months prior to the current month, which did not afford the opportunity to adjust lower lags or the current month. However, this model provided an indication of expected MRSA infection levels (the probability of exceeding the 70th percentile) three months in advance. The hospital therefore has 1–3 months to mitigate the risk of increased MRSA infections through increased vigilance, patient protocols, and increased hygiene. Table 4 shows months in which we can pre-determine the expected MRSA level given fluoroquinolone and co-amoxiclav use in the preceding 3-month window. We note that the predicted probabilities for these future months were based on co-amoxiclav use being below and fluoroquinolone use being above identified thresholds. The alert signal for both January 2022 and March 2022 is predicted to be Medium, highlighting the need to increase vigilance and attempt to suppress risk via increased antimicrobial stewardship and infection control practices, hygiene, and additional patient protocol measures.

### 2.5. Recommendations for Selected Antibiotic Use for Management of Both MRSA and ESBL-Producing E. coli [36]

Based on this study and our previous work [36], co-amoxiclav, fluoroquinolones, and third-generation cephalosporins were identified as being associated with the incidence of MRSA and ESBL-producing *E. coli.* Therefore, these antibiotics should be subjected to close monitoring and antimicrobial stewardship interventions to influence their use. It is possible to evaluate the identified antibiotic thresholds for various pathogens within the combined modelling framework and thus develop recommendations for antibiotic guidelines. For example, to control ESBL-producing *E. coli*, the threshold for fluoroquinolones was determined in a previous study to be 61.14 DDD/1000 OBD with a 95% confidence range of (55.96 to 68.27 DDD/1000 OBD) and with a one-month lag effect on the ESBL-producing *E. coli* response [36]. In the MRSA threshold logistic model used in this paper, the threshold for fluoroquinolones was 55.96 DDD/1000 OBD with a 95% confidence range of (37.37 to 75.15 DDD/1000 OBD) and a three-month lag effect on MRSA response. In this case, the lowest identified threshold for both the ESBL-producing *E. coli* and MRSA models (i.e., 55.96 DDD/1000 OBD) should be considered as target for antimicrobial stewardship approaches.

In December 2021, fluoroquinolone use was 75.15 DDD/1000 OBD, above the 61.14 DDD/1000 OBD threshold for the ESBL-producing *E. coli* model [36]. It produced a Medium ESBL-producing *E. coli* alert signal in January 2022 [36]. Prior information regarding the impact of fluoroquinolones that increased the ESBL-producing *E. coli* incidence rate was known in November 2021 and efforts could have been made to bring this down to under 55.96 DDD/1000 OBD. Through this mitigation effort, the ESBL-producing *E. coli* alert signal would be downgraded from Medium to Low in December 2021. We would also know that the planned reduction in fluoroquinolones for December 2021 impacts the MRSA alert signal for March 2022. By keeping fluoroquinolones under 55.96 DDD/1000 OBD, the MRSA alert signal would also have been reduced from Medium to Low. This allows for anticipatory control of risk outcome, months in advance, by evaluating the impact of fluoroquinolone usage in the current month as it affects the increased (or decreased) probability of the MRSA incidence rate three months later. Without knowledge of the relational impact of fluoroquinolone usage on MRSA incidence rates, and considering the March 2022 result (Table 4), the probability of a high MRSA incidence rate is 28.30%, which corresponds to a Medium alert signal. However, with prior knowledge of this modelled relationship, if fluoroquinolone usage was controlled under 55.96 DDD/1000 OBD in December 2021, the probability of a high MRSA incidence rate can be reduced to 13.44%. It therefore lends itself well to policy management as it offers anticipatory control of high pathogen incidence rates.

## 3. Discussion

In our recently published work, we developed a modelling concept named threshold logistic [36]. We examined the utility of this approach using a Gram-negative pathogen, namely, ESBL-producing *E. coli* [36]. In this study, we applied threshold logistic modelling methods to understand the impact of exceeding thresholds of antibiotic use that result in increasing the incidence rate of the Gram-positive MRSA pathogen in the same population. We wanted to provide an example of identifying antibiotic use targets for the management of prevalent Gram-positive and -negative pathogens in hospitals. The benefit is an opportunity for the hospital to engage in actionable counter-measures to mitigate accelerated rates of AMR.

Through applying the threshold logistic modelling approach, we: (i) determined the cut-off percentile value of MRSA incidence that defines a critical level of MRSA; (ii) enabled the identification of thresholds for fluoroquinolone and co-amoxiclav use that would accelerate MRSA incidence rates and increase the probability of reaching critical levels; (iii) achieved a better understanding of the effect of antibiotic use on the probability of reaching the defined critical level of MRSA incidence when antibiotic usage exceeds an identified threshold; (iv) provided a near real-time performance monitoring feedback system through a scorecard approach; (v) provided risk scores/alert signals when antibiotic use exceeded critical levels through what-if scenarios; and (vi) provided overall recommendations for the management of AMR based on the findings of this study and our recently published work [36].

The importance of thresholds analysis lies in its ability to provide quantitative targets to inform antimicrobial stewardship by providing thresholds that should not be exceeded in order to control pathogen incidence rates. This approach has been reported in a number of studies using different pathogens and study sites [1,19,20,35]. In a recent evaluation, the authors identified different thresholds for the use of antibiotics, with respect to the same pathogen, across participating hospitals, indicating the need for tailored analysis based on modelling data from each hospital [35]. The analysis undertaken in these studies was performed on a continuous outcome variable, i.e., the pathogen incidence rate [19,20,35]. Thresholds identified in modelling ‘continuous outcomes’ represent the start of an observed increase in the assessed pathogen’s incidence rate; they are not indicative of the pathogen’s incidence rate exceeding tolerable levels or occurring outside normal variation of historical incidence rates. It is important to emphasize, as has been shown by our published work [36], that the progressive increase in levels of antibiotic use above the identified threshold will be associated with an increased probability of the pathogen’s incidence rate being outside its normal variation. This suggests the need to define a critical level of pathogen incidence rate, and to predict the probability of reaching this defined critical level of incidence when antibiotic usage exceeds an identified threshold.

The critical level of pathogen incidence rate is used as the binary event required for threshold logistic regression modelling methods. In a hospital setting, the critical level of the pathogen incidence rate may be based on a hospital mandate, a tolerance level set by government recommendation, or on empirical analysis of historical infection rates.

For the purposes of this study, we used empirical analysis of historical MRSA incidence rates to define when a critical level of MRSA incidence rate was reached. The 70th percentile (0.276 cases/1000 OBD) of MRSA incidence was selected as the cut-off value defining a high incidence rate (Figure 2). Fluoroquinolones were found to have a threshold at 55.96 DDD/1000 OBD, and co-amoxiclav was found to have a threshold at 312.19 DDD/1000 OBD. Using antibiotics above these thresholds would accelerate MRSA incidence rates and increase the probability of reaching the defined critical levels. We also produced a predicted probability of exceeding the 70th percentile of the historical MRSA incidence rate. Probabilities ranged from near zero when antibiotics were close to the thresholds, to approaching a near certain probability (≥99%) as antibiotic use increased above the thresholds. This was shown in the contour plot (Figure 5), which can be used to understand the risk of antibiotic use when exceeding recommended thresholds.

Logistic regression provides a risk score or probability of an event occurring relative to an overall set of covariates. The threshold logistic concept combines the benefits of identifying individual antibiotic use that should be kept below certain thresholds and the ability to generate risk scores or probabilities of pathogen incidence rates exceeding a critical level. In the present work, we provided a sample scorecard which can be used for near real-time feedback on the effectiveness of implemented antibiotic policies, for example, keeping antibiotic use below identified thresholds. In addition, the prediction utility offered the opportunity to perform “what-if scenarios” using different levels of antibiotic use to evaluate the expected impact on predicted pathogen incidence in the following months [36]. In this study, our analysis identified both antibiotics (i.e., fluoroquinolones and co-amoxiclav) with a 3-month lag, therefore, they were entered into the model three months prior to the current month. This does not permit adjusting earlier lags with the intent of affecting the predicted MRSA outcome. However, the model can predict the probability of approaching the 70th percentile of MRSA incidence three months in advance (i.e., January–March 2022 in this worked example). This provides hospital policy makers the opportunity to mitigate the risk of increased MRSA infection through several auxiliary methods, e.g., increased vigilance, patient treatment protocols, and increased infection control practices, as well as hand and environmental hygiene.

Finally, and in part (2.5) of the Results section, we aimed to provide an example of how to identify antibiotics at high risk of driving antibiotic resistance in hospitals, and of how we can combine the results from modelling such pathogens to inform effective antimicrobial stewardship. In our previous study, we modelled the relationship between certain antibiotic classes and ESBL-producing *E. coli* (a Gram-negative pathogen) [36]. Using the same hospital study site and the same population, we modelled the relationship between certain antibiotic classes and MRSA (a Gram-positive pathogen). These were the two most frequently-occurring pathogens, which enabled the conduct of robust statistical analysis. Based on the findings for both pathogens, we made our recommendations as follows: (i) there is a need to monitor/intervene and influence the use of co-amoxiclav, fluoroquinolones, and third-generation cephalosporins; (ii) for fluoroquinolones, and since use was associated with both MRSA and ESBL-producing *E. coli* with different thresholds, the lowest identified threshold (i.e., 55.96 DDD/1000 OBD) should be considered as the target for antimicrobial stewardship; and (iii) the ‘what-if scenarios’ should be used to predict incidences for future months, and to inform antimicrobial stewardship accordingly. In relation to the latter, we provided an example of how a reduction of antibiotic use for ESBL-producing *E. coli* (determined in our previous study [36]) may also reduce the probability of a high MRSA incidence rate in future months, downgrading the alert signal from Medium to Low in March 2022. Importantly, if mitigation measures are taken to control pathogen incidence rates, then evaluations of the various actions can be measured in the hope of finding optimal mitigation actions. Since we are using predictive modelling to influence future pathogen incidence rates, threshold models should be regularly re-estimated to adjust for evolutionary changes in the data.

We used robust statistical methods to routinely analyse the collected data for all adult inpatients, therefore selection and information bias are unlikely. Nevertheless, it was not possible to adjust for potential changes in the patient population or for case mix. The estimated model can be improved via the inclusion of further explanatory variables, for example, infection prevention and control activities and proxy measures for changes in patient population and case mix, if possible [37,38]. During the study period, no significant changes to hand hygiene or cleaning practices were made. The effect of COVID-19 on the model was considered and found to be non-significant. Finally, this work represented a single-centre assessment, therefore, the study would benefit from a multi-centre assessment.

In conclusion, we developed an innovative method, i.e., the threshold logistic modelling concept, to improve our understanding of the effect of antibiotic use on antibiotic resistance when usage exceeds recommended threshold levels, with the utility of providing quantitative thresholds to inform effective antimicrobial stewardship and provide a near real-time performance monitoring feedback system for policy assessment and for keeping antibiotic use below identified thresholds. Setting targets for antibiotic use through identifying the relevant thresholds has the benefit of avoiding wholesale restriction of antibiotics and associated challenges [24], along with providing access to antibiotic treatment. The threshold logistic modelling approach can help in defining critical pathogen incidence rates and facilitate a coded alert signal (High, Medium, or Low risk) for predicting probability. Future research should work toward implementing and evaluating the proposed approach prospectively in hospitals, with the aim of determining the best counter-measures to mitigate the risk of increased resistant pathogen incidence in hospitals.

## 4. Methods

### 4.1. Study Design and Population

The work was conducted at Pinderfields Hospital (700 beds), Mid Yorkshire Hospitals NHS Trust in West Yorkshire, England. The Trust cares for 500,000 people, providing medical and surgical services, intensive care, haematology/oncology, a regional burns unit, a regional spinal injuries unit, and community services. All adult inpatients admitted to Pinderfields Hospital were included in the study. Retrospective data collection was performed for the study period from January 2015 to December 2021. In relation to the analytical methods employed in this study, the minimum requirement was 5 years of monthly antibiotic use and microbiology data [19,20]. In this study, 7 years of data were used, based on the availability of the longest period of consistent antibiotic use and MRSA data.

We hypothesized that the use of third-generation cephalosporins, fluoroquinolones, macrolides, and co-amoxiclav could explain variations in the incidence of MRSA. These antibiotics were identified a priori based on their available resistance profiles obtained from the hospital microbiology department (which showed that MRSA isolates were resistant to ciprofloxacin, clarithromycin, and erythromycin in 66.9%, 63.3%, and 63.4% of the cases, respectively), along with published evidence of their role as risk factors for driving hospital MRSA incidence rates [24,25,26,27,28,29,30,31].

### 4.2. Microbiology and Pharmacy Data

An MRSA case was defined as any adult inpatient (≥18 years), who was admitted to Pinderfields Hospital between 1 January 2015 and 31 December 2021, and who had a positive MRSA result during their admission. Data were obtained using an infection control software (ICNET; Clinical Surveillance Software’ NG 1.7.1.0, Baxter International INC, UK). Duplicates were excluded if they were within 30 days of hospital readmittance. All pink colonies on the MRSA-selective agar plate were tested using a latex agglutination kit for *Staphylococcus aureus* identification. If the latex result was unclear, identification was confirmed using MALDI-TOF mass spectrometry. Any isolates identified as *Staphylococcus aureus* were subjected to sensitivity testing against a range of antibiotics to identify MRSA strains. In line with the European Committee on Antimicrobial Susceptibility Testing (EUCAST) guidelines, sensitivity testing was performed by seeding a sensitivity plate with a suspension of the organism in a specified dilution and adding antibiotic discs of known concentration to the plates. Following incubation at 34–36 °C for 16–20 h, a zone of inhibition around a disc was indicative of resistance. The antibiotic used to determine whether an isolate was MRSA was cefoxitin.

Antibiotic usage quantities were obtained on a monthly basis from the hospital pharmacy information systems (JAC). These data were then converted into Defined Daily Doses (DDD), in line with the classification of antimicrobials for systemic use (J01) in the WHO/ATC index and expressed as DDD per 1000 occupied bed-days (OBD) [39,40].

### 4.3. Modelling and Statistical Analysis

Antibiotic and MRSA series were initially assessed using descriptive statistics, plots, and an examination of the cross-correlation functions of the series to identify linear lag structures and relationships. Non-linear relationships and thresholds identification in antibiotic use that influenced MRSA incidence rates were then explored with Multivariate Adaptive Regression Splines (MARS) methods and other non-linear value-segmenting models [41,42,43,44]. For the application of a logistic approach, the continuous pathogen rate was converted into a binary event [45]. The binary event was defined as a critical level of the pathogen incidence rate that was set through exploratory numerical methods.

#### 4.3.1. Defining a Critical Level of Pathogen Incidence Rate

Continuous MRSA incidence rates were initially analysed in relation to individual antibiotic usage variables at various lags using MARS and non-linear value-segmentation threshold methods to identify candidate drivers of increased MRSA incidence rates. The possibility for any shift that may have occurred due to COVID-19 after March 2020 was evaluated through the inclusion of a binary indicator variable. For the purpose of identifying the critical level of the incidence rate that produced the highest classification accuracy, we partitioned the MRSA incidence rate by various percentiles, specifically between the 50th and 85th percentiles of the incidence rate of MRSA. We then employed a search algorithm that recursively evaluated all threshold combinations of the candidate antibiotic series at various lags against the binary classifier. The 50th–85th percentiles were selected as a target since this target would be defined in the upper 50% of the historical data and would include enough observations so as not to be considered a rare event. The 70th percentile (0.276 cases/1000 OBD) was identified as the cut-off for the binary event as it produced a dominant classification performance in both classification accuracy and number of modelled results in the top decile of accuracy measures. The binary event is defined as

Event = Ifelse (MRSA incidence rate ≥ 0.276, 1, 0).

#### 4.3.2. Threshold Logistic Method

In relation to the binary event, we employed a threshold logistic search algorithm that jointly optimized:

- the critical level of the MRSA incidence rate between the historical 50th and 85th percentile range;
- the lag structure of the antibiotic series;
- antibiotic threshold values.

The threshold logistic method [46,47] can be expressed as

$$Pr\left(Y_{i}=y|x1_{i},\;x2_{i},\;\ldots ,\;xm_{i}\right)=\left\{\begin{array}{c} p_{i}\;\;\;\;\;\;\;\;\;\;\;\;\;\;\;\;\;if\;y=1\\ 1-p_{i}\;\;\;\;\;\;\;\;\;if\;y=0 \end{array}\right.$$

where *x*1_*i*_ through *x*m_*i*_ represent the threshold-adjusted and lag-adjusted antibiotic explanatory variables.

Classification accuracy, using a probability cut-off that maximized the sum of sensitivity and specificity, was used for model selection. This was extracted from the detailed model summary information exported from the iterative estimation results of the search algorithm. The computed area under the curve (AUC) of the receiver operator characteristic (ROC) curve was used as a confirmatory measure of classification power [48,49]. A one-at-a-time (OAT) approach was employed to undertake sensitivity analysis of the lower and upper limit around the optimized threshold value. The 70th percentile was confirmed to be of highest accuracy for class separation.

Predicted probabilities (risk scores) of the threshold logistic regression model were generated. A coded alert signal (High, Medium, or Low risk) was created based on the MinMax transformation of the predicted probabilities (risk score) coming from the threshold logistic model.

$$z=\frac{prob-\min\left(prob\right)}{\max\left(prob\right)-\min\left(prob\right)}$$

To maximize the overall distribution accuracy of a Low signal classifying an infection rate as being below the 70th percentile and a High signal classifying an infection rate as being greater than the 70th percentile, we computed the cut-off ranks using a linear programming (LP) technique. To define Low, Medium, and High coded alert signals, the cut-off ranks optimized through LP were 0.0 to ≤0.42, >0.42 to <0.69, and ≥0.69 to 1.0, respectively. The SCA Statistical System version 8.2 (Scientific Computing Associates Corp., River Forest, Illinois, USA) and R software version 4.1.0 (R Foundation for Statistical Computing, Vienna, Austria) were used to perform analysis.

## Figures and Tables

**Table 1:** Threshold logistic results in modelling the MRSA incidence rate at the 70th percentile, January 2015 to December 2021.

Predictor Variable	Lag	Median Use (IQR)	Threshold (95% Confidence Limit) *	Relation to Threshold	Coefficient (95% CI)	*p*-Value	Odds Ratio (95% CI)
Constant	NA	NA	NA	NA	−1.862 (−2.717 to 1.008)	<0.001	0.1553 (0.07 to 0.37)
Fluoroquinolones use (DDD/1000 OBD)	3	64.55 (53.58–76.39)	55.96 (37.37 to 75.15)	Above	0.0486 (0.012 to 0.085)	0.0099	1.0498 (1.01 to 1.09)
Co-amoxiclav use (DDD/1000 OBD)	3	270.90 (247.3–297.0)	312.19 (213.72 to 333.16)	Above	0.0493 (0.010 to 0.089)	0.0139	1.0505 (1.01 to 1.09)

* 95% confidence limit around the optimized threshold value, which was derived using a one-at-a-time (OAT) approach; IQR, interquartile range; NA, not applicable.

**Table 2:** Risk scores for MRSA incidence rates exceeding the 70th percentile for 2021.

Date	MRSA Rate Observed above 70th Percentile (0.276 Cases/1000 OBD)	Fluoroquinolones Use (DDD/1000 OBD) at Lag 3 (Threshold-Adjusted)	Co-Amoxiclav Use (DDD/1000 OBD) at Lag 3 (Threshold-Adjusted)	Predicted Probability MRSA above 70th Percentile	Coded Alert Signal
January	Above	2.29	0.00	0.148	Low
February	Below	3.72	0.00	0.157	Low
March	Below	0.00	0.00	0.134	Low
April	Below	0.00	0.00	0.134	Low
May	Below	0.00	0.00	0.134	Low
June	Below	37.39	0.00	0.489	High
July	Below	12.88	0.00	0.225	Medium
August	Below	0.00	0.00	0.134	Low
September	Below	3.86	0.00	0.158	Low
October	Below	15.25	0.00	0.246	Medium
November	Above	0.00	0.00	0.134	Low
December	Below	1.13	0.00	0.141	Low

**Table 3:** Summary of numbers of coded alert signals when the MRSA incidence rate observed was above and below the 70th percentile (January 2015–December 2021).

		MRSA Observed above or below 70th Percentile (0.276 Cases/1000 OBD))	
		Above	Below
Coded Alert Signal	Low	5	28 (5.6:1)
	Medium	6	15
	High	13 (1:1)	13

**Table 4:** Three-month-ahead alert signals based on threshold logistic model.

Date	Fluoroquinolones Use (DDD/1000 OBD) at Lag 3 *	Co-Amoxiclav Use (DDD/1000 OBD) at Lag 3 *	Predicted Probability MRSA above 70th Percentile	Coded Alert Signal
**January 2022**	73.76	213.72	0.269	Medium
**February 2022**	66.40	233.59	0.205	Low
**March 2022**	75.15	273.90	0.283	Medium

* Fluoroquinolones threshold = 55.96 DDD/1000 OBD; co-amoxiclav threshold = 312.19 DDD/1000 OBD.

## Data Availability

The data are contained in the article.
